# Running from stress: a perspective on the potential benefits of exercise-induced small extracellular vesicles for individuals with major depressive disorder

**DOI:** 10.3389/fmolb.2023.1154872

**Published:** 2023-06-15

**Authors:** Reine Khoury, Corina Nagy

**Affiliations:** ^1^ Integrated Program in Neuroscience, McGill University, Montreal, QC, Canada; ^2^ McGill Group for Suicide Studies, Douglas Mental Health University Institute, Verdun, QC, Canada; ^3^ Department of Psychiatry, McGill University, Montreal, QC, Canada

**Keywords:** aerobic exercise, small extracellular vesicles, major depressive disorder, miRNA, epigenetics, sex differences, blood-brain barrier

## Abstract

Aerobic exercise promotes beneficial effects in the brain including increased synaptic plasticity and neurogenesis and regulates neuroinflammation and stress response via the hypothalamic-pituitary-adrenal axis. Exercise can have therapeutic effects for numerous brain-related pathologies, including major depressive disorder (MDD). Beneficial effects of aerobic exercise are thought to be mediated through the release of “exerkines” including metabolites, proteins, nucleic acids, and hormones that communicate between the brain and periphery. While the specific mechanisms underlying the positive effects of aerobic exercise on MDD have not been fully elucidated, the evidence suggests that exercise may exert a direct or indirect influence on the brain via small extracellular vesicles which have been shown to transport signaling molecules including “exerkines” between cells and across the blood-brain barrier (BBB). sEVs are released by most cell types, found in numerous biofluids, and capable of crossing the BBB. sEVs have been associated with numerous brain-related functions including neuronal stress response, cell-cell communication, as well as those affected by exercise like synaptic plasticity and neurogenesis. In addition to known exerkines, they are loaded with other modulatory cargo such as microRNA (miRNA), an epigenetic regulator that regulates gene expression levels. How exercise-induced sEVs mediate exercise dependent improvements in MDD is unknown. Here, we perform a thorough survey of the current literature to elucidate the potential role of sEVs in the context of neurobiological changes seen with exercise and depression by summarizing studies on exercise and MDD, exercise and sEVs, and finally, sEVs as they relate to MDD. Moreover, we describe the links between peripheral sEV levels and their potential for infiltration into the brain. While literature suggests that aerobic exercise is protective against the development of mood disorders, there remains a scarcity of data on the therapeutic effects of exercise. Recent studies have shown that aerobic exercise does not appear to influence sEV size, but rather influence their concentration and cargo. These molecules have been independently implicated in numerous neuropsychiatric disorders. Taken together, these studies suggest that concentration of sEVs are increased post exercise, and they may contain specifically packaged protective cargo representing a novel therapeutic for MDD.

## Introduction

Major depressive disorder (MDD) is one of the leading causes of disability worldwide (Smith, 2014). The World Health Organization estimates more than 300 million people worldwide suffer from MDD (Soares et al., 2021). Depressive disorders are characterized by mood changes, anhedonia, disrupted sleep, cognitive impairments, and suicidal thoughts (Dean and Keshavan, 2017). Without efficient treatments, depression is likely to be associated with several other physical comorbidities such as cancer, stroke, and acute coronary syndrome (Kang et al., 2015). Current treatments rely on antidepressant drugs, however only 30%–40% of depressed patients achieve a complete remission with treatment, and unremitted patients are at a higher risk for suicide (Block and Nemeroff, 2014). Given that traditional treatments are inadequate and have several pharmacological side effects (Alemi et al., 2021), there is an urgent need to better characterize molecular pathways involved in MDD to find novel therapeutic targets. Furthermore, the global prevalence of MDD is twice as common in women than in men (Albert, 2015). This differential risk arises from a combination of physiological, psychological, and environmental, including cultural factors, with sex as a biological variable (SABV) as a central factor (Salk et al., 2017). However, the underlying neurobiological mechanisms are not fully understood, and more research is needed to characterize the biological sex differences in MDD for better and more targeted treatment options (Bangasser and Cuarenta, 2021).

Exercise is one of the most effective non-pharmacological therapeutic means to sustain a healthy mind (Cotman et al., 2007; Jaworska et al., 2019), and has been used as a treatment for 26 various illnesses including depression, anxiety, and schizophrenia (Pedersen and Saltin, 2015).

Physical exercise (PE) is classified into aerobic and anaerobic PE depending on the level of intensity, the types of muscle fibers used, and the recruited metabolic energy system (CCA, 2017). Aerobic exercise in particular has been linked to several mental health benefits (Paluska and Schwenk, 2000). It rescues many features of neuropsychiatric disorders (Gujral et al., 2017), reduces depression and anxiety (Belvederi Murri et al., 2019), promotes hippocampal plasticity (Cooper et al., 2018), increases learning and memory (Hötting and Röder, 2013), and delays the onset of neurodegenerative symptoms (Valenzuela et al., 2020). Strong evidence shows that aerobic exercise is an effective treatment for depression (Kvam et al., 2016; Mandolesi et al., 2018), and the American Psychiatric Association recommends it as a non-pharmacological antidepressant treatment. However, the neural molecular mechanisms responsible for exercise-induced benefits on depressive symptoms are elusive. Since MDD, like other complex disorders, has numerous causal factors, and treatments are not equally effective in all patients, there has been increased interest in individualized treatments in psychiatry (Rush et al., 2006). Consequently, understanding the peripheral and central effects of exercise, and their interplay, will be an asset for pinpointing personalized treatment strategies. Interestingly, some of the beneficial effects of exercise are mediated by the release of extracellular vesicles (EVs) into circulation (Nederveen et al., 2021). Extracellular vesicles are released by nearly all cells in the body and carry molecular cargo that can influence the recipient cell. Many different molecules have been associated with EVs including, DNA, RNA, proteins, lipids, and microRNAs (miRNAs), the latter an epigenetic regulator whose dysregulation has been associated with many disease states including depression (Smalheiser et al., 2014; Belzeaux et al., 2017; Saeedi et al., 2021). EVs are found in numerous biofluids and are capable of crossing the blood-brain barrier which positions them as strong potential biomarkers, particularly for psychiatric phenotypes (Saeedi et al., 2021). Mounting evidence suggests that EVs, and particularly their cargo play a role in numerous brain-related functions including synaptic plasticity, neuronal stress response, cell-to-cell communication, and neurogenesis (Lafourcade et al., 2016; Luarte et al., 2017; Saeedi et al., 2019).

Recent studies show that after a single bout of aerobic physical activity, skeletal muscles release EVs into circulation (Frühbeis et al., 2015) that are thought to be taken up by other tissues, including the brain (Chen et al., 2016; Vechetti et al., 2021). An interesting demonstration of the ongoing interactions between the central nervous system and the periphery is the ability to isolate neuronal-origin EVs from plasma (Mustapic et al., 2017). Likewise, there is reason to believe peripheral EVs can exert their effects centrally (Shi et al., 2019). As such, there is increasing interest in the role of EVs as drivers of inter-organ communication associated with exercise, specifically through the transport of molecular signals. Given that, both exercise and EVs are shown to have a mediating role in mental illnesses, and the known link between exercise and EV release, we posit that exercise-induced EV release mediates the effect of PE on MDD.

In this perspective review, we demonstrate the potential of aerobic exercise as an adjunct treatment option for major depression. We delve into the mechanisms by which exercise affects brain health, including its impact on synaptic plasticity, neuroinflammation, and monoamine neurotransmission. While other manuscripts have focused on the triad of exercise, EVs, and depression (Soares et al., 2021), here, we expand on this triad by providing additional perspectives on the therapeutic effects of exercise factors and skeletal muscle-released small EVs (sEVs) on MDD. We explore multiple mechanisms beyond inflammation, shedding the light on other potential pathways implicated in the therapeutic effects of exercise-induced sEVs. Specifically, we highlight the potential role of exercise-induced sEVs in promoting neural plasticity, and neurogenesis, drawing insights from a range of both animal and human studies.

Moreover, we emphasize the significance of sex as a crucial biological variable in understanding the interplay between exercise, sEVs, and MDD. Most importantly, our review takes an additional step in this novel field by exploring the different mechanisms underlying the passage of sEVs across the BBB, going beyond the current focus on exercise, sEVs, and MDD. This discussion aims to provide a more nuanced understanding of the relationship between exercise and sEV-mediated effects on the nervous system.

## Physical exercise in alleviating symptoms of major depression

Research into the beneficial effects of exercise on depression dates to the early 1900s, with studies by Franz and Hamilton (1905), Vaux (1926), and Ström-Olsen (1937). Paffenbarger et al. (1994) conducted a study on 31,000 Harvard College graduates aged 35–74 years, following them for 23–27 years. The study revealed that physically active individuals (walking, climbing, descending stairs, etc.) and sports players had 17%–28% lower incidence of depression compared to their sedentary counterparts (Paffenbarger et al., 1994). Similarly, a recent study on 5,877 participants aged 15–54 years found that regular exercise was associated with a 25% decrease in the likelihood of major depressive disorder (Goodwin, 2003). Additionally, exercise has been found to have a protective effect against depression regardless of age or geographical location, as reported in a study by Schuch et al. (2018). Indeed, prospective surveys suggest that exercise is protective against MDD, however, the causality and direction of this relationship are not explicit. Nevertheless, a genome-wide association study (GWAS) from 600,000 adults showed that people with genetic markers associated with MDD (44 independent genome-wide significant SNPs) were not less likely to exercise (objective accelerometer-based physical activity), hence MDD does not make one intrinsically less likely to exercise (Choi et al., 2019).

The exercise regimens used in research vary widely across studies leading to inconsistent findings or interpretations of the data. Nonetheless, there have been numerous studies looking at the therapeutic role of aerobic exercise in the context of MDD which have provided consistent, comparable, and measurable outcomes (Carter et al., 2015; Jaworska et al., 2019; Morres et al., 2019). Aerobic exercise can be defined by aerobic metabolism, the use of large muscle groups, prolonged duration, and consistent rhythm. Patients with MDD engaging in aerobic treadmill training 3 times a week for 16 weeks at home or in a supervised setting to exhaustion were found to have reduced Hamilton Depression Rating Scales (HAMD) scores similar to effects observed in patients treated with antidepressants (Blumenthal et al., 2007). Similarly, inactive and unmedicated 18–24 year olds with depression who underwent a 12-week program of aerobic exercise (3x/week; 1 h/session) showed an increased cardiovascular score and approximately 50% decreased depression HAMD scores (Jaworska et al., 2019). A systematic review and meta-analysis on the dose-response relationship between PE and depression suggests that small doses of aerobic exercise (2.5 h/week of brisk walking) are sufficient to lower risks of depression (Pearce et al., 2022), and several other studies have demonstrated that in some cases it is as effective as antidepressants (Netz, 2017). However, in severely depressed patients receiving pharmacologic therapy, exercise is considered as an adjunct treatment along with cognitive-behavioral therapies for managing symptoms of depression (Craft and Perna, 2004). Nonetheless, Stanton and colleagues showed that the use of moderate-intensity aerobic exercise three times a week for a minimum of 9 weeks could lower the symptoms of depression in adults aged 18–65 years with a diagnosis of depression (Stanton and Reaburn, 2014). Moreover, Knubben and colleagues showed that patients diagnosed with MDD may quickly show a significant improvement in their mood: the Bech-Rafaelsen Melancholy Scale (BMRS) scores dropped by 36% after 10 days of endurance exercise that consisted of daily walking on a treadmill (Knubben et al., 2007).

Exercise-induced benefits on MDD are most commonly associated with certain neuro-molecular mechanisms including; increasing the levels of serotonin and norepinephrine (Ross et al., 2023), increasing the expression of neurotrophins such as brain-derived neurotrophic factor (Russo-Neustadt et al., 2000), regulating the hypothalamic-pituitary-adrenal (HPA) axis activity (Lopresti et al., 2013), as well as reducing inflammation (Paolucci et al., 2018). The beneficial effects of exercise are thought to be mediated through the release of “exerkines” which encompass multiple signaling factors including metabolites, proteins, nucleic acids, and hormones that crosstalk between the brain and the periphery (Lee et al., 2019; Stephan and Sleiman, 2021; Chow et al., 2022). Various exercise-induced factors released into the bloodstream from muscle, liver, and bone, have been shown to cross the blood-brain barrier (BBB) enhancing brain-derived neurotrophic factor (BDNF) signaling, and alleviating the symptoms of depression (Sleiman et al., 2016; Karnib et al., 2019; Rentz et al., 2020). Additionally, aerobic exercise regulates miRNA expression in the prefrontal cortex and the hippocampus (Fernandes et al., 2018; Qu et al., 2020). Many mechanisms and pathways related to depression are influenced by exerkine related-factors of which, many are speculated to be transported in circulation in sEVs supporting the potential cross-talk between peripheral organs or between peripheral organs and the central nervous (Safdar and Tarnopolsky, 2018).

There have been many excellent reviews focusing on the efficacy of different exercise variables such as intensity (Rethorst et al., 2013), duration and frequency (Pearce et al., 2022) in the context of exercise and depression. These broader factors are outside the scope of this review as we will focus on changes in small extracellular vesicles (sEVs) characteristics during exercise and their impact on depression.

### The effect of exercise on synaptic plasticity, neuroinflammation, and the monoamine neurotransmitters

Synaptic plasticity is shown to be disrupted in the hippocampi of individuals with depression (Liu et al., 2017). Interestingly, exercise positively affects brain function via the induction of synaptic plasticity (Mandolesi et al., 2017). Studies on humans and animals using magnetic resonance imaging (MRI) have shown that prolonged voluntary exercise increases the volume of the hippocampal region in mice (Islam et al., 2020), and long-term moderate-intensity exercise increases the volume of several brain regions in humans (Nauer et al., 2020). By triggering several different systems, such as the inflammatory system, the monoamine system, the neurotrophic pathway, and the HPA axis, exercise can mediate its favorable effects on the brain. Among these systems, the brain-derived neurotrophic factor (BDNF) signaling pathways is one of the most significant mechanisms activated by PE. BDNF is a protein that promotes the growth and survival of neurons (Miranda et al., 2019). *Postmortem* human studies of individuals with depression and/or who have died by suicide show reduced BDNF protein levels in the hippocampus and other brain regions, contrary to individuals receiving antidepressant treatment (Chen et al., 2001; Duman and Monteggia, 2006; Kim et al., 2007). In humans, aerobic exercise training triggers a rise in serum BDNF levels, which is linked to the increase of hippocampal volume, enhancement of spatial memory (Erickson et al., 2011; Khoury et al., 2023), and a reduction of depressive symptoms (Erickson et al., 2012). However, exercise-induced neuronal plasticity is inhibited in mice with the single nucleotide polymorphism (SNP) in the BDNF gene Val66Met (BDNF^Met/Met^) (Hariri et al., 2003), or when the BDNF signaling is blocked (Vaynman et al., 2004).

**FIGURE 1:**
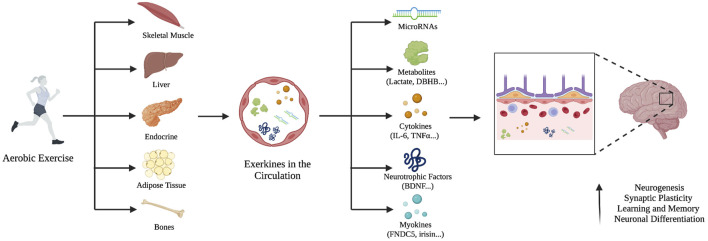
The systemic release of exerkines and their effect on the brain. Aerobic exercise stimulates different peripheral tissues to release exerkines into the bloodstream such as microRNAs, metabolites, cytokines, neurotrophic factors, and myokines. They can cross the blood-brain barrier, increase neurogenesis, improve synaptic plasticity, and neural differentiation.

It has been demonstrated that crosstalk between the periphery and the brain can be triggered by exercise resulting from the release of molecules and factors from the liver, muscles, and bones into the bloodstream to support neuronal plasticity (Stephan & Sleiman, 2021) (Figure 1). These exercise-induced factors activate convergent neural pathways, with BDNF signaling in the central role of this coordinated response (Fakhoury et al., 2022). Upon exercise, the muscle secretes several factors that activate BDNF signaling and regulate neural plasticity such as the myokine *fibronectin type III domain-containing protein 5* (FNDC5) (Wrann et al., 2013), and lactate (El Hayek et al., 2019).

Moderate to vigorous exercise increases lactate production and accumulation in the muscle and enhances the uptake of skeletal muscle-derived lactate by the brain, where it exerts its function in several ways (Xue et al., 2022). Exercise-induced lactate is transported to the hippocampus via the bloodstream where it binds to monocarboxylate transporters (MCT 1-4s), modulates hippocampal class I histone deacetylase (HDAC2/3) levels, which results in reduced depressive-like symptoms in mice (Proia et al., 2016; Karnib et al., 2019). It has also been suggested that lactate activates G Protein-Coupled Receptor 81 (GPR81) (Lauritzen et al., 2014), to promote anti-inflammatory effects (Hoque et al., 2014), and inhibit GABAergic transmission (Bozzo et al., 2013). Lactate can also increase the expression levels of the lysine deacetylase Sirtuin 1 (SIRT1), to activate the peroxisome proliferator-activated receptor gamma coactivator 1-alpha and the estrogen related receptor alpha complex (PGC1alpha/ERRa), causing an increase in the expression levels of FNDC5 in the hippocampus (Wrann et al., 2013). FNDC5 and its cleavage product irisin activate BDNF transcription via intermediate pathways, enhancing learning and memory formation (El Hayek et al., 2019). Four weeks of moderate physical exercise in adult female mice raised hippocampal FNDC5/irisin levels and elicited an antidepressant like effect in these mice (Gruhn et al., 2021). The same treadmill training protocol promoted hippocampal cell proliferation, neuronal differentiation, and cell survival, while increasing hippocampal levels of FNDC5 of female mice (Siteneski et al., 2020). However, again BDNF SNP Val66Met (BDNF^Met/Met^) inhibits the beneficial effects of exercise-induced increase in the hippocampal mRNA levels of FNDC5 and BDNF (Ieraci et al., 2016). A recent review suggests that the disruption of the endogenous BDNF activity such as the Val66Met BDNF variant potentiates sensitivity to stress and stress-inducible disorders. The authors argue regulators of BDNF may orchestrate sensitivity to stress, and trauma (Notaras and van den Buuse, 2020). For example, in mice exposed to chronic social defeat stress, overexpression of the PGC-1 alpha/FNDC5/BDNF signaling pathway in skeletal muscles contributes to mice’s resilience to stress (Zhan et al., 2018). Interestingly, exercise also causes bones to secrete osteocalcin, an endocrine hormone that activates the G Protein-Coupled Receptor, GPR158, increasing the levels of irisin, and in turn, BDNF in the brain (Khrimian et al., 2017; Nicolini et al., 2020). Of note, the genetic deletion of osteocalcin leads to increased depressive-like behaviors in mice (Oury et al., 2013).

Inflammatory processes are also involved in the complex interplay of pathways conferring PE’s positive effects on depression. Numerous lines of clinical and translational research demonstrate the link between depression and heightened immune activation through increased numbers of proinflammatory cytokines such as IL-6, and TNF-α (Raison et al., 2006; Nobis et al., 2020). On the other hand, physical exercise has been shown to reduce pro-inflammatory responses through increased expression of the transcriptional activator PGC1 alpha (Peroxisome proliferator-activated receptor gamma coactivator 1-alpha) (Handschin and Spiegelman, 2008). In line with this, it was demonstrated that moderate-intensity exercise can lower the proinflammatory cytokine TNF- α blood levels, and reduce depression scores (Paolucci et al., 2018).

Another hypothesized system to benefit from the effects of exercise on MDD is monoamines. Historically, the monoaminergic system was targeted for treatment interventions as it was consistently found that a depressed state was caused by an insufficient supply of the monoamine neurotransmitters serotonin, norepinephrine, and dopamine in the central nervous system (Boku et al., 2018). Exercise is known to restore the levels of these three main monoamine neurotransmitters dopamine, norepinephrine, and serotonin (Lin and Kuo, 2013), hence contributing to exercise’s anti-depressive effects.

### Possible sex differences in exercise efficacy on brain health

Few studies on exercise and brain health have specifically addressed sex differences, and it is still not known whether the postulated mechanisms underlying the beneficial effects of exercise on the CNS differ between sexes. Barha and colleagues conducted a systematic review on aerobic exercise and found that the positive effects on cognitive functions, including decision making, were directly related to the number of female participants in the study. This suggests that certain beneficial outcomes of exercise are sex-specific (Barha et al., 2017). Similarly, a controlled trial by Baker and colleagues found major sex differences in the response to aerobic exercise in older adults with mild cognitive impairment (MCI). Specifically, the results revealed that females in the aerobic exercise group had increased BDNF serum levels and exhibited significant improvements in cognitive function compared to the control group. To the contrary, there were no significant changes in BDNF levels or cognitive function in males within the aerobic exercise group. These sex differences imply that aerobic exercise have a more positive impact on cognitive performance in females with MCI compared to males (Baker et al., 2010). As previously mentioned, aerobic exercise improves brain health by enhancing neural plasticity, and hippocampal neurogenesis, which is also influenced by sex differences. For example, one study found that adult female and male rodents show differences in neurogenesis following spatial training. Male rats outperformed female rats in the spatial test, and the training increased the quantity of BrdU-labeled cells in male rats but not in female rats (Chow et al., 2013). Furthermore, BDNF protein levels vary during the estrus cycle in rodents and the menstrual cycle in humans, as higher estradiol levels are associated with higher BDNF expression. BDNF and estrogen have been linked to various neurological and psychiatric disorders, which may have an impact on sex differences and disorders such as addiction (Harte-Hargrove et al., 2013). Indeed, a randomized controlled trial showed that a 6-month aerobic exercise training leads to higher serum levels of BDNF in females aged 55 years and older with mild cognitive impairment but not in males of the same age range (Barha et al., 2017). This suggests that the physiological response to aerobic training is not the same in males and females, making it crucial to move beyond the “one size fits all” approach and consider sex differences in exercise efficacy in developing efficient and personalized exercise interventions to promote a healthy brain.

The influence of exercise on these various biological processes lends support to the neuroprotective and anti-depressive properties of exercise. As such, exercise represents an important potential mediator of depression and future research should focus on the interactions of “exerkines” with the known underlying neurobiology of major depression.

### The role of extracellular vesicles in the systemic adaptation to exercise

As described above, exercise’s health benefits are partially attributed to the plethora of bioactive molecules released of exerkines or exercise factors into the bloodstream. Aside from the traditional peptide secretion pathway, little is known about how these factors are delivered in circulation from their tissue of origin to close-by or distal destinations. Extracellular vesicles represent a possible delivery mechanism as they have been found to carry exerkine-type molecules including different RNA species, proteins, and metabolites (Chow et al., 2022). Extracellular vesicles can be classified into different subpopulations based on their biogenesis, and properties such as size, protein composition, miRNA content, and cellular origin (van Niel et al., 2018). The three main types of EVs are: small extracellular vesicles (sEVs), microvesicles, and apoptotic bodies (Gheinani et al., 2018). sEVs (40–200 nm) are the smallest population of EVs, they originate from the inward luminal budding of the membrane, followed by the formation of multivesicular bodies and fusion with the plasma membrane which leads to the release of these sEVs into the extracellular space (Lee et al., 2012). Microvesicles (50–1,000 nm) originate from the direct budding off the plasma membrane, to deliver cargo to recipient cells (Lee et al., 2012). Finally, apoptotic bodies (500–2000 nm), are membrane-bound vesicles that originate from the disassembly of apoptotic cells (Xu et al., 2019).

With just less than a decade of research exploring the relationship between exercise and sEVs, the field is still in its infancy, and we are only starting to elucidate its role in exercise. However, skeletal muscle has been extensively studied as an endocrine organ, and it is becoming increasingly clear that skeletal muscles release myokines and exerkines (IL-6, IL-15, myostatin, BDNF, and muscle-specific microRNAs) in response to muscle contractions (Vechetti et al., 2021). There is evidence that exercise’s beneficial effects on mood are partially mediated through skeletal muscle-brain communication via myokines such as IL-1β, IL-6, TNF-α, and IL-10 (cytokines released by muscle tissue) (Mucher et al., 2021). Exercise-induced myokines are taken up by other tissues including the brain where they regulate several functions, including mood, memory, locomotion, and neuronal injury protection, demonstrating muscle-brain cross-talk (Scisciola et al., 2021). Following different high and moderate intensity exercise protocols, it was demonstrated that moderate intensity exercise can temporarily elicit both pro- and anti-inflammatory temporary responses in the blood (Cerqueira et al., 2019). For example, in otherwise healthy college students, moderate-intensity exercise was optimal for improving depressive symptoms which appears to be mediated through a decrease in TNF-α levels in the blood (Paolucci et al., 2018). On the other hand, in individuals with treatment-resistant depression, a rise in TNF-α levels was positively correlated with the antidepressant effect of a 12-week aerobic exercise (Rethorst et al., 2013).

Recently, the mechanisms through which skeletal muscle operates as an endocrine organ have been broadened to include sEVs (Rome et al., 2019). *In vivo* research on sEVs originating from skeletal muscle is challenging due to the current inability to specifically label and monitor sEVs released from this tissue. However, multiple *in vitro* studies have clearly shown that myoblasts and myotubes release proteins using sEVs (Forterre et al., 2014; Choi et al., 2016). Muscle-derived sEVs have been shown to serve as a cell-free therapeutic approach for muscle regeneration, a biological cue for stem cell differentiation (Choi et al., 2016), as well as, a modulator of metabolism (Takafuji et al., 2020).

In the context of EV cargo, D’souza and colleagues demonstrated that specific populations of miRNAs from sEVs in muscles, plasma, and circulation (Table 1) increased significantly in ten healthy young men (18–35 years old) following high-intensity exercise in the form of cycling (D’Souza et al., 2018). Likewise, comparing acute exercise in active vs. inactive men on a cycle ergometer for 40 min, resulted in 7 differentially expressed miRNAs immediately post-exercise and 8 miRNAs 3 h post-exercise. The miRNA signature post-acute exercise was shown to be correlated with the activation of IGF-1 signaling in the exercise group (Nair et al., 2020). In rats, acute aerobic treadmill training for 40 min/day for 1 week resulted in the differential expression of twelve miRNAs and a concurrent rise in serum sEV concentration (Oliveira et al., 2018).

**TABLE 1:** Effects of physical exercise on circulating extracellular vesicles in humans.

Study	Subjects	Physical exercise	Extracellular vesicles
Frühbeis et al. (2015)	Healthy male subjects (*n* = 12, age = 41.1 ± 14.9)	1-Cycling ergometer incremental test starting at 50 W and increasing power by 50 W every 3 min until exhaustion. 2- Treadmill running incremental test starting speed at 6 km/h and increasing velocity by 2 km/h every 3 min with a constant incline of 1.5% until exhaustion.	Cycling
			⇑ 2.7-fold NTA in small EVs count (Post-Ex)
			⇑ 5.2-fold in Flot1and Hsp/Hsc70 (Post-Ex)
		Type: Acute endurance	Running
			⇑ 1.5-fold NTA in small EVs (Post-Ex)
			⇑ Flot1 (Post-Ex)
D’Souza et al. (2018)	Healthy male subjects (*n* = 10, age = 24.6 ± 4.0)	Cycling at peak power output - 10 × 60s intervals with 75-s rests between intervals.	⇑ target miRNA (Post-Ex)
		Type: High-intensity interval training (HIIT)—acute aerobic	⇑ Muscle-, plasma-, and exosome-responsive miRNAs: miR-1-3p, -16-5p and 222-3p
			⇑ Muscle and plasma responsive miRNAs: miR-23a-3p, 208a-3p and -150-5p
			⇑ Muscle and exosome responsive miRNAs: miR- 486-5p, 378a-5p, 126-3p
			⇑ Exosome responsive miRNAs: miR- 23b-3p, 451a and 186-5p
Whitham et al. (2018)	Healthy male subjects (*n* = 11)	Cycling ergometer test for 1h - 30 min at 55%, 20 min at 70% and 10 min or until exhaustion at 80% of VO_2_ max.	⇑ 2-fold NTA in small EVs count (Post-Ex)
		Type: Acute endurance	⇔ NTA small EVs count (4 h Post-Ex)
			⇑ 322 proteins isolated from EV (Post-Ex)
Lansford et al. (2016)	Healthy male and female subjects (Male: *n* = 16 M, age = 24.5 ± 0.8//Female: *n* = 10, age = 22.40 ± 0.52)	Cycling ergometer test at 150–200 W increasing by 25 W every 2 min until exhaustion.	⇑ activated endothelial-derived vesicles in males by 107% (Post-Ex)
		Type: Acute endurance	⇑ Endothelial progenitor cell-derived vesicles in females by 253% (Post-Ex)
Helmig et al. (2015)	Healthy male subjects (*n* = 5)	Treadmill running incremental test. 1.5% inclination and starting with 6 km/h and increasing by 2 km/h every 3 min until exhaustion.	⇑ Flot1and Hsp/Hsc70 (90 min Post-Ex)
		Type: Acute endurance	
Brahmer et al. (2019)	Healthy male athletes (*n* = 21, age =	Cycling incremental test starting at 40 W, increasing by 40 W every 3 min until exhaustion.	⇔ NTA count of multiple EV subtypes
		Type: Acute endurance	⇑ SEC- EVs during exercise
			⇑ CD63, CD9, CD81
Bei et al. (2017)	Healthy male and female subjects (*n* = 16, age = 54 ± 11)	Exercise stress test	⇑ CD63 (Post-Ex)
		Type: Acute aerobic	⇑ release of circulating EVs (Post-Ex)
Karvinen et al. (2020)	Healthy male and female subjects (Males *n* = 3, age = 28.7 ± 5.7//Females *n* = 5, age = 24.8 ± 6.1)	Cycling ergometer incremental test at maximal aerobic capacity (VO_2_max)	⇑ miR-21 levels in sweat EVs (Post-Ex)
		Type: Acute endurance	

In a very intriguing study by Bei and colleagues, exercise-induced sEVs were injected into sedentary mice at an exercise-relevant concentration resulting in a significant reduction in the infarct size *in vivo*, and cardiomyocyte apoptosis *in vitro*. The authors found an enhancement in the cardioprotective effect against acute ischemia/reperfusion injury in mice driven by sEV (Bei et al., 2017). Another study showed that intravenous injection of exercise-induced sEVs reduced tumor volume by 35% in a rat model of metastatic prostate cancer (Sadovska et al., 2022). Several other studies show promise for the use of extracellular vesicles in clinical applications both as biomarkers and as therapeutic delivery vehicles (O’Brien et al., 2020). According to a study by Pierdoná and colleagues, obese and sedentary adolescents (15–16 years old) that respond to acute aerobic exercise produce larger-sized plasma EVs with a higher protein yield (Pierdoná et al., 2022).

Future treatments for obesity and type 2 diabetes mellitus (T2DM) may use modified small extracellular vesicles loaded with exerkines, which have been shown to reduce obesity and T2DM and positively affect metabolic health (Safdar et al., 2016). These results confirm the notion that aerobic exercise does indeed influence miRNA expression in sEVs, and that sEVs play a key role in mediating the systemic beneficial effects of exercise. However, more studies investigating exercise-related factors such as intensity, type, and frequency, are needed for a better understanding of the link between exercise and sEVs.

The data summarized in Table 1 and Table 2 represent recent trials evaluating the circulating EV patterns following physical exercise in humans, and rodents, respectively. Nanoparticle tracking analysis (NTA) of plasma EVs showed increased concentration in human male subjects that underwent incremental cycle ergometry, intense treadmill running. Post-exercise plasma analysis showed a 5.2-fold surge in sEV markers: FLOT1 and HSP/Hsc70 (Frühbeis et al., 2015). Additionally, an exercise stress test caused a rapid increase in plasma EVs in human subjects (Bei et al., 2017).

**TABLE 2:** Effects of physical exercise on circulating extracellular vesicles in rodents.

Study	Sample	Physical exercise	Extracellular vesicles
Barcellos et al. (2020)	Male Wistar rats (2- and 22-month-old)	Forced treadmill running at 60% of VO_2_max, Climbing ladder of 85° inclination, and acrobatic exercise, combined. (6 min of aerobic training, 6 min of acrobatic training and 6 min of resistance training)	⇑ BDNF, IL-1β, CD63 in EVs only from aerobic exercise aged rats. ß IL-1β in EVs from acrobatic and combined exercise adult rats
		Duration: 20 min x 3x/week x 12 weeks	
		Type: Aerobic, Resistance, Acrobatic	
Ma et al. (2018)	Male C57BL/6J mice (8–10-week-old)	Forced treadmill training at low intensity at 5 m.min^-1^ or moderate intensity at 10 m min^-1^ for 60 min/d, 5 days/week	⇑ 2-fold, and 4-fold increase in endothelial progenitor derived exosomes in low intensity and moderate intensity exercise
		Duration: 60 min/d x 5 days/week x 4 weeks	
		Type: Aerobic	
Bertoldi et al. (2018)	Male Wistar rats (3-, 21-, and 26-month-old)	Moderate aerobic forced treadmill exercise	⇑ 10% CD63 levels 18 h Post-Exercise
		Duration: 20 min/day x 2 weeks	
		Type: Aerobic	
Bei et al. (2017)	Male C57BL/6J mice (8-week-old)	Swimming (Day 1: 5 min twice a day, increase by 10 per day until 90 min)	⇑ 1.85-fold NTA in serum EVs
		Duration: Incremental until 90 min, 2x/day x 3 weeks	
		Type: Aerobic	
Castaño et al. (2020)	Male C57BL/6J mice (15-week-old)	High intensity interval training (incremental until exhaustion)	⇑ miR-133a, miR-133b, and miR-206 in circulating exosomes
		Duration: 5 weeks	
		Type: Anaerobic	

**FIGURE 2:**
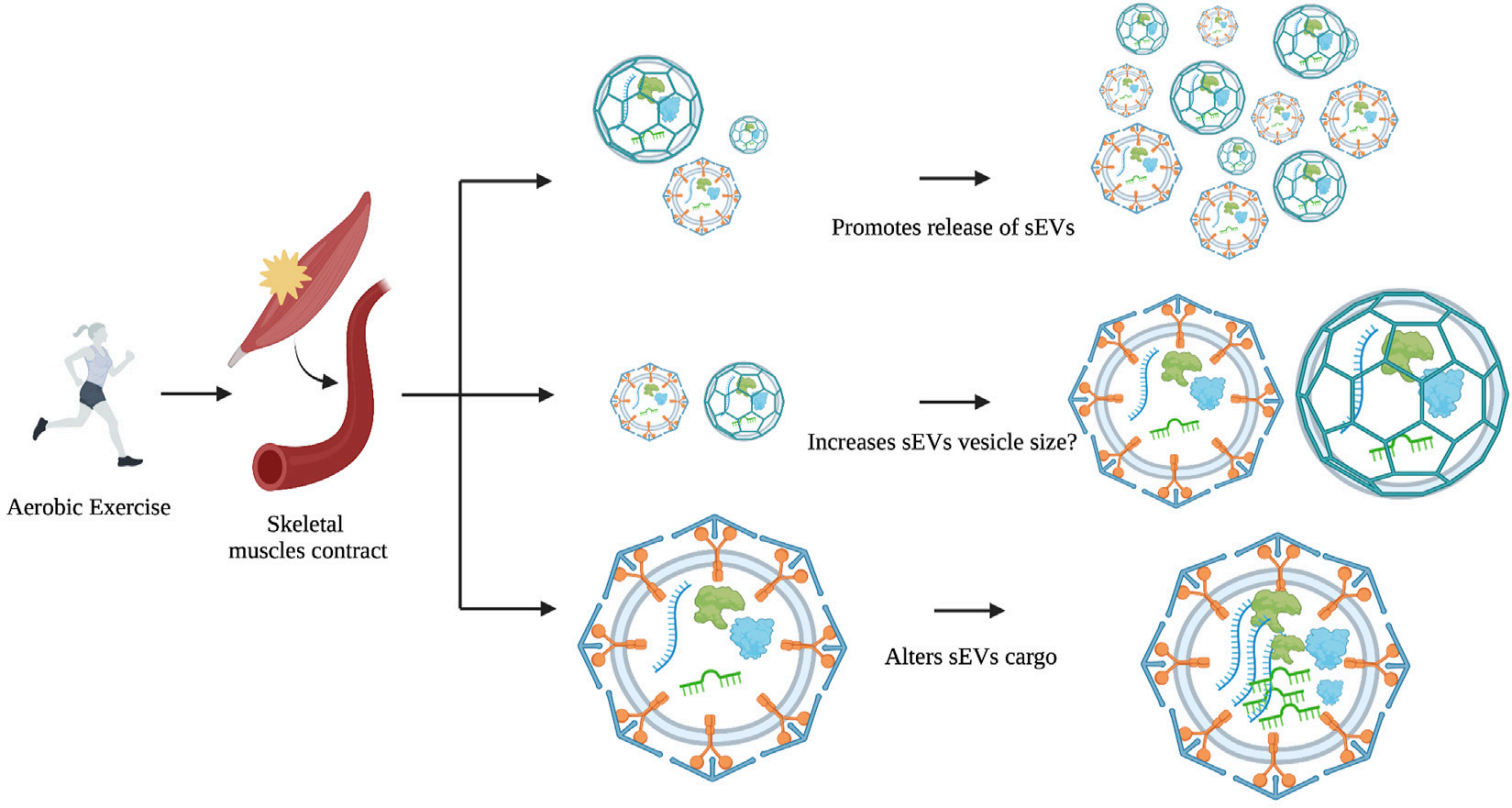
The possible effects of exercise on EV size, concentration, and cargo. During or after physical exercise, skeletal muscles release sEVs into the bloodstream. Evidence to date suggests that exercise is more likely to affect cargo and concentration and not size.

However, a similar study showed no significant alterations in NTA particle size or number following a cycling ergometer test in the blood; but there was an increase in several sEV markers including CD9, CD63, and CD81 (Brahmer et al., 2019). In animal studies, aerobic exercise causes an increase in BDNF and CD63 EV levels but does not alter EV size or concentration in aged rats (Barcellos et al., 2020). Acute aerobic treadmill exercise in rats was found to increase EV concentration in the blood and alter miRNA cargo that targets the MAPK pathway (Oliveira et al., 2018). Likewise, a systemic analysis of 19 *in vivo* and *ex vivo* studies concluded that aerobic exercise increases sEV markers (Alix, CD63, CD81, and Flot-1), and stimulates their release into circulation without changing its vesicle size or concentration (Estébanez et al., 2021). These results imply that physical exercise has more influence on sEV content and concentration, rather than size (Figure 2).

## Extracellular vesicles and major depressive disorder

Circulating EVs and particularly the smaller subtype, have garnered a lot of attention as a potential diagnostic tool in psychiatric disorders (Kano et al., 2019). Intercellular communication mediated by sEVs has been linked to neurogenesis (Luarte et al., 2017), neuronal plasticity (Lafourcade et al., 2016), neuroinflammation (Dalvi et al., 2017), and transcriptional regulation (Ung et al., 2014). Alterations to the above-mentioned neurobiological processes have previously been implicated in MDD, which has led researchers to hypothesize that sEVs might be involved in the pathology of MDD. Multiple studies suggest that sEVs carry miRNAs throughout the body (Tian et al., 2017; Liu et al., 2019; Munir et al., 2020), and most of the miRNA targets are associated with different pathologic pathways in depression (Gruzdev et al., 2019).

### The effect of sEVs on neurogenesis

While still controversial, some studies have shown that adult hippocampal neurogenesis (AHN) is disrupted in individuals with depression (Kim & Park, 2021) with studies pointing to specific miRNAs as contributors to this disruption (Ortega et al., 2021). In this context, certain miRNAs found in sEVs released by astrocytes were found to be elevated (miR-9, miR-26b, mi-29a, miR-125b, miR-145, and miR-543) and others, downregulated (miR-26a, miR-221), in stressful circumstances. Notably, these miRNAs have been implicated in regulating neuronal differentiation and proliferation by targeting different molecular targets such as Notch signaling genes, and BMP/TGFβ signaling genes (Luarte et al., 2017). Likewise, others have shown BDNF, VEGF, Notch1, miR-124, and miR-9 — molecules known to regulate neurogenesis—have been identified as cargo present in sEVs from various peripheral tissues (Zhang and Xu, 2022). Serum and sEV BDNF levels measured by ELISA were found to be decreased in depressed patients compared to healthy controls (Dwivedi, 2009; Gelle et al., 2021). Intranasal administration of EVs from neural stem cells (NSCs) derived from human induced pluripotent stem cells into adult rats and mice resulted in the incorporation of EVs by neurons, microglia, and astrocytes in all brain regions and promoted hippocampal neurogenesis via several enriched proteins such as Agrin and pentraxin-3 (Upadhya et al., 2020). This suggests that proteins from EVs play a role in increasing neurogenesis, which might be disrupted in individuals with MDD (Saeedi et al., 2019). On the other hand, injecting sEVs containing amyloid precursor protein, a cargo considered to be pathogenic, into the dentate gyrus of mice, weakened adult hippocampal neurogenesis (Zheng et al., 2017). Genome-wide miRNA profiling of blood-derived sEVs from individuals with MDD and healthy controls showed an upregulation of miR-139-5p expression in peripheral sEVs in individuals with depression. Injection of a miR-139-5p antagomir rescued depressive-like symptoms and increased hippocampal neurogenesis in stressed mice (Wei et al., 2020). Serum sEV-derived miR-139-5p was found to be one of the most differentially expressed miRNAs between MDD and healthy patients by Liang and colleagues (Liang et al., 2020). Inhibition of miR-139-5p plays an antidepressant-like role by enhancing the levels of nuclear receptor subfamily 3 group C member 1 (NRC1), BDNF, and the phosphorylated/total tyrosine kinase receptor B (p-TrKB/TrKB) (Su et al., 2022). Taken together, these studies suggests that sEVs are implicated in promoting or inhibiting adult hippocampal neurogenesis, and thus, potentially influencing the depressive state.

### The effect of sEVs on synaptic plasticity

sEVs released from depolarized neurons have been found to regulate synaptic plasticity. For example, proteomic analysis of neuron-derived sEVs found the synaptic-plasticity-associated protein MAP1b to be enriched in these vesicles (Goldie et al., 2014). Specifically, proteins such as transmembrane protein proline-rich 7 (PRR7) are secreted by neurons by sEVs, and are taken up by a recipient cell, resulting in the elimination of excitatory synapses and maintaining normal synapse count (Huo et al., 2021). Neuronal sEVs have been shown to influence synaptic pruning by microglia by increasing the microglial expression of complement component 3 (Bahrini et al., 2015). These findings support sEVs as regulators of synaptic pruning, hence influencing synaptic plasticity.

### The effect of sEVs on inflammation

There is evidence that sEVs are regulators of neuroinflammation, for example, monocytes activated by interferon-alpha secrete sEVs carrying altered miRNA profiles, subsequently activating the nuclear factor-κB, and increasing the inflammatory response (Dalvi et al., 2017). Likewise, microglia cells can release sEVs that carry IL-1- β, caspase-1, and P2X7 receptors which induce neuroinflammation (Frühbeis et al., 2013). sEV protein cargo from human cerebral microvascular endothelial cell culture is changed in response to the inflammatory cytokine tumor necrosis factor (TNF) (Dozio & Sanchez, 2017). Using serum sEVs from immune-challenged mice, recipient animals showed increased microglial activation with augmented CNS expression of pro-inflammatory cytokine mRNA and associated miRNA (Li et al., 2018). Li and colleagues demonstrated that natural killer (NK) cell derived sEVs carry miR-207 that rescued depressive-like behaviors in mice through the targeting of TLR4-interactor with leucine-rich repeats (Tril) and by decreasing the levels of IL-1β, IL-6, and TNF-α released by astrocytic cells in culture (Li et al., 2020). Several studies have also demonstrated the relationship between sEVs and neuroinflammation in depression (Li et al., 2020; Wang et al., 2021; Duarte-Silva et al., 2022; Xie et al., 2023). Protein profiling of peripherally sourced neural-derived sEVs using ELISA revealed higher levels of IL34/CD81 in MDD compared to the healthy controls (Kuwano et al., 2018).

Gut inflammation has been related to the development of various mental diseases, including anxiety and depression (Clapp et al., 2017). Recently, the microbiome has gained considerable attention for its relationship to neuropsychiatric disorders including depression. For example, Rhee and colleagues, found that MDD patients carry more DNA from specific bacteria genera (*Prevotella* 2 and *Ruminococcaceae* UCG-002) in their bacteria-derived sEVs from serum (Rhee et al., 2020).

In a review article by Soares et al. (2021), the researchers propose a captivating hypothesis suggesting the effects of exercise on MDD are mediated through altered concentration and cargo profiles of circulating extracellular vesicles capable of modifying inflammatory signals throughout the body. The authors examine clinical studies focusing only on the relationship between PE and MDD, EVs and MDD, as well as PE and EVs. Additionally, they present a wide range of potential biomarkers, including brain-derived biomarkers (GFAP, α-synuclein, glutamine synthetase), exerkines (myostatin, myonectin), neurotrophic factors (BDNF, nerve growth factor (NGF)), miRNAs, and stress and inflammation signaling mediators (interleukins/cytokines, C reactive protein (CRP)), that hold promise in the field of exercise and depression research (Soares et al., 2021).

### The journey of sEVs from peripheral tissues to the brain

sEVs must cross the vascular BBB to functionally communicate between the CNS and the periphery. The exact molecular mechanisms regulating EV passage across the BBB remain unclear.

sEVs follow a complex journey from being secreted by parental cells in the periphery to crossing the blood-brain barrier and infiltrating into the brain (Matsumoto et al., 2017). Understanding EV trafficking is important for elucidating the underlying crosstalk between tissues integral to exercise’s beneficial effects on MDD. Table 1 shows that different aerobic exercise protocols induce a change in EV cargo in the bloodstream, and/or to a lesser extent, the rapid increase in EV concentration (Table 1). In the brain, the concentration of sEVs significantly increases after exercise (Zhang et al., 2021), either due to increased passage through the blood-brain barrier or as a direct consequence of exercise on the brain and local release of EVs. The exact mechanisms involved in transporting sEVs across the BBB are not yet fully understood. However recent research has provided some evidence showing that sEVs can indeed cross the BBB (Fowler, 2019). For example, Alvarez-Erviti et al. demonstrated that intravenously injected sEVs can deliver short interfering RNA to the brain in mice, specifically targeting neurons, microglia, and oligodendrocytes, leading to a specific gene knockdown (Alvarez-Erviti et al., 2011). Different *in vitro* models have been used to investigate the mechanism of sEV migration through the BBB (Ramos-Zaldívar et al., 2022). Using human brain microvascular endothelial cells (BMECs) transwell assays, Chen et al. (2016) showed that luciferase carrying sEVs have the ability to cross the BMEC layer under inflamed conditions. Morad et al. (2019) demonstrated that cancer-derived EVs can breach the BBB *in vivo* through transcytosis (across the interior of the cell) via decreasing the expression of rab7 in brain endothelial cells and enhancing their transport.

*In vivo* and *in vitro* studies have shown that peripheral sEVs interact with the BMECs of the BBB under stroke-like conditions, and enter the brain via different mechanisms such as: micropinocytosis, receptor-mediated transcytosis, and adsorptive-transcytosis (Chen et al., 2016; Heidarzadeh et al., 2021). Morad et al. (2019) monitored the distribution of brain-seeking EVs (Br-EVs) in a zebrafish model to explore the transcytosis mechanism in the brain via live imaging. Br-EVs were taken up by cells of the brain parenchyma, showing that Br-EVs can cross the BBB. Time-lapse imaging also showed movement of these vesicles towards the plasma membrane where it fused with the membrane, indicative of a transcytosis mechanism (Morad et al., 2019).

**FIGURE 3:**
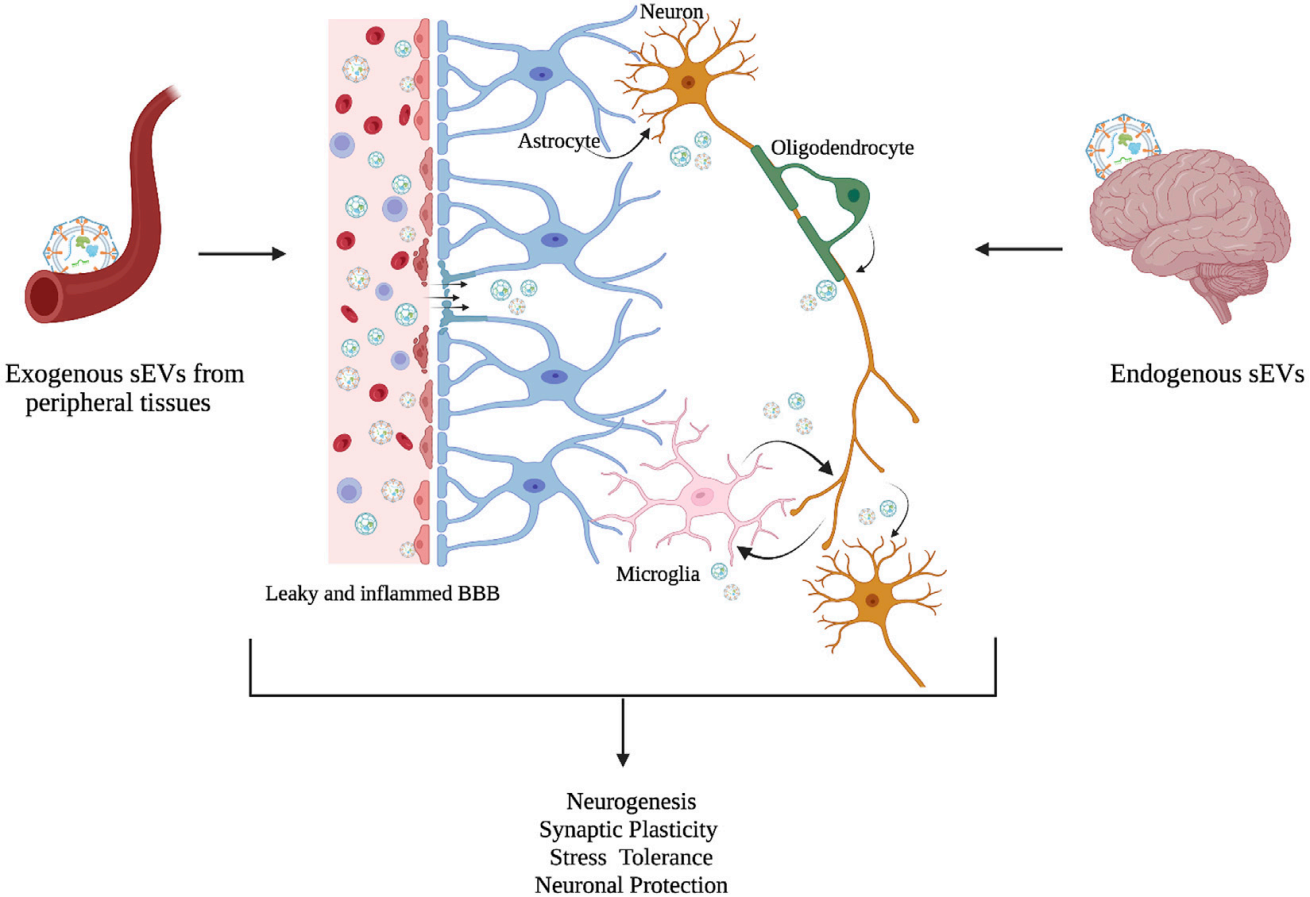
sEVs as intercellular mediators between the periphery and the brain. Although the molecular mechanism is not fully understood, an increasing number of studies suggest that exogenous sEVs from peripheral tissues cross BBB, and act as intercellular communicators, along with endogenous sEVs to regulate physiological processes in the brain such as neurogenesis, synaptic plasticity, stress tolerance, and neuronal protection. Changes in these physiological processes have been associated in MDD. A leaky and inflamed BBB is linked with MDD pathology in the brain (Adapted from Saeedi et al., 2019).

Given that, crossing the blood-brain barrier facilitates the connection between the brain and the periphery, sEVs play a critical role in some of the systemic alterations reported in neuropsychiatric disorders (Saeedi et al., 2019). In individuals with MDD, the BBB is disrupted, and neuroinflammation has been proposed as the potential cause of this disruption (Wu et al., 2022) (Figure 3). There is a significant increase in the infiltration of cells into the brain of individuals with MDD as cytokines increase the BBB’s permeability (Cheng et al., 2018). Interestingly, inflammation of the BBB has been demonstrated to increase EV passage by five folds (Chen et al., 2016). However, sEVs are not just a connection between the periphery and the brain in neuropsychiatric and neurological disorders, they also provide information on the changing processes of the CNS through brain-derived EVs present in circulation. For instance, glioblastoma-specific mRNA has been discovered in circulating sEVs and has been proposed as a diagnostic biomarker (Skog et al., 2008). Several studies have found alterations in sEV cargo, particularly miRNAs in individuals with MDD that target neuroinflammation, synaptic plasticity, and neurogenesis. Dysregulation of various miRNAs such as miR-223, miR-451, miR-182, and miR-16 is linked to MDD (Gururajan et al., 2016), supporting the role of sEVs as intercellular communicators, and potential mediators in the pathogenesis of neuropsychiatric disorders such as depression.

More research is needed to fully understand the underlying mechanisms of the bi-directional transfer of sEVs across the BBB, particularly as brain-derived sEVs may reflect physiological changes in psychiatric disorders.

## The link between physical exercise, small extracellular vesicles, and major depressive disorder

**FIGURE 4:**
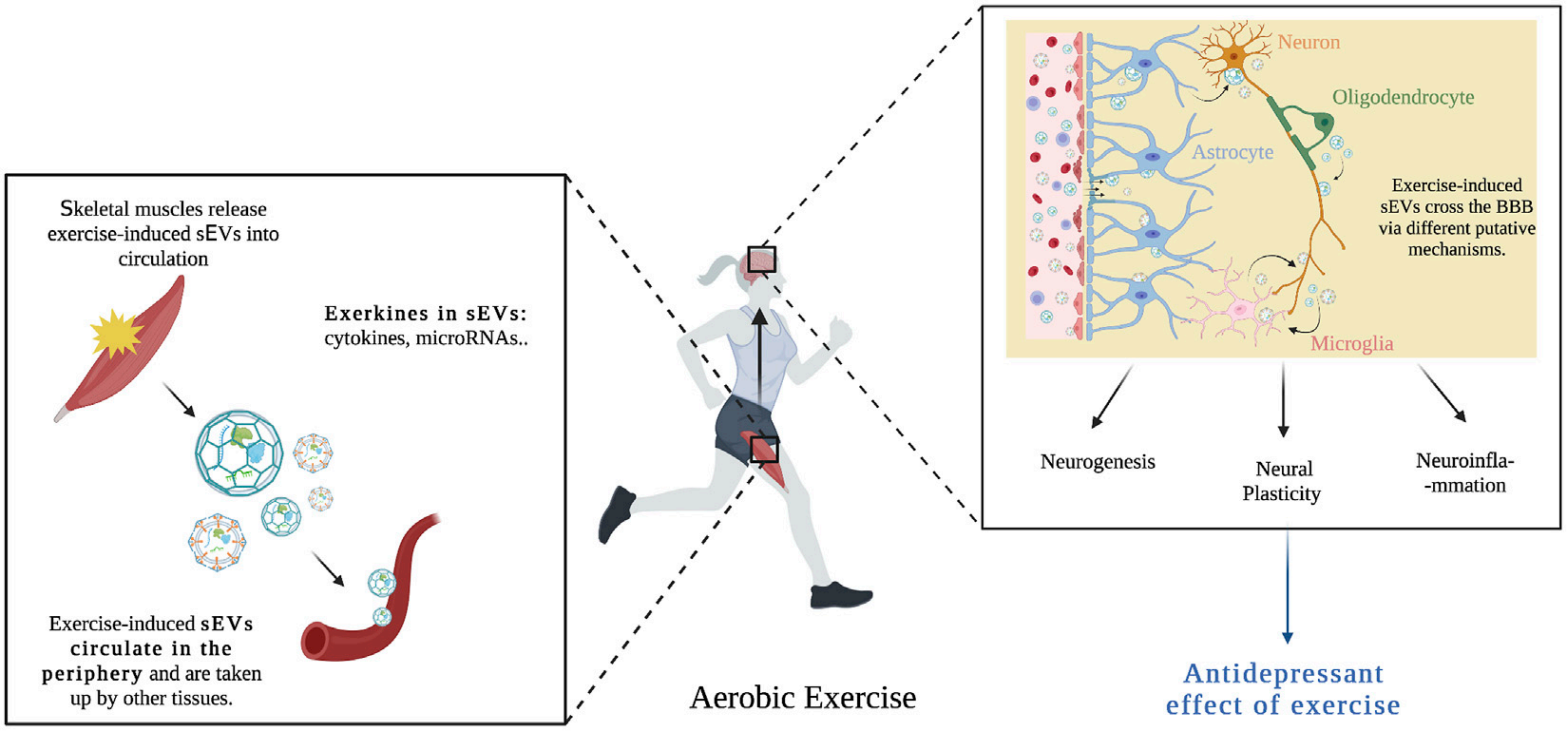
Aerobic exercise causes skeletal muscles to release exercise-induced sEVs into blood circulation. These sEVs contain important exerkines (cytokines, microRNAs) and are taken up by other tissues such as the brain. Exercise-induced sEVs cross the BBB via different putative mechanisms (transcytosis, micropinocytosis, and clathrin-dependent endocytosis), and influence neural pathways associated with the etiopathogenesis of major depression, such as neurogenesis, neural plasticity and neuroinflammation, hence mediating the beneficial effects of exercise on MDD.

Exercise has been identified as an effective preventative and treatment strategy for a variety of neuropsychiatric disorders (Smith and Merwin, 2021). However, the use of exercise as a treatment remains elusive due to the paucity of information on the underlying neurobiological processes, and the lack of knowledge on the metabolic crosstalk between exercise-induced peripheral changes and the brain in MDD. Current literature suggests that aerobic exercise can ease symptoms of depression via several neural mechanisms (Xie et al., 2021), as well as modulate circulating sEVs and their RNA and protein content which is important for intercellular communication for exercise-induced adaptations (Nederveen et al., 2021). Exercise might increase plasma sEV levels by releasing them from peripheral tissues such as skeletal muscles or change its cargo signature, principally RNA and protein (Bei et al., 2017; Oliveira et al., 2018). Subsequently, exercise-induced sEVs, circulate to other tissues such as the brain to regulate specific cell responses by increasing nerve cell growth, regulating inflammation (Fuller et al., 2020), and possibly modulating depressive symptoms in individuals with MDD (Figure 4). The cargo of circulating extracellular vesicles post-exercise include different exerkines such as: miRNA (miR-139-5p, miR-206, and miR-207), neurotrophic factors (BDNF, VEGF, TrkA, and TrkB), and cytokines (IL-1β, IL-6, and TNF-α). Identifying and assessing these markers in circulating sEVs may provide a more comprehensive view of the signaling mechanisms necessary for the beneficial effects of exercise on MDD (Table 3). Although there is no research showing the role of exercise-induced sEVs in the antidepressant effects of exercise, the combined evidence presented here indeed supports the possibility of this relationship. Taken together, these studies suggest that the beneficial effects of exercise on MDD can be mediated by sEVs through their capacity to regulate processes such as synaptic plasticity, neuroinflammation, and neurogenesis.

**TABLE 3:** Potential role of exerkines encapsulated in small EVs and their relationship with major depressive disorder.

Exerkines in sEVs		Relationship with exercise	Role in major depressive disorder
miRNAs	miR-451a	A single bout of high intensity interval training (cycling at peak power output - 10 × 60s intervals) increases the expression of miR-451a in exosomes D’Souza et al. (2018)	Serum levels of miR-451a are lower in depressed patients (Kuang et al., 2018)
	miR-150-5p	A single bout of high intensity interval training (Cycling at peak power output - 10 × 60s intervals) increases the expression of miR-150-5p in muscles and exosomes D’Souza et al. (2018)	Lower levels of miR-150-5p are linked to higher levels of prenatal discomfort (Foley et al., 2023). Additionally, miR-150-5p knockout in mice, results in an increase in anxiety-like behavior Zhang et al. (2021)
Neutrophins	Brain-derived neurotrophic factor (BDNF)	Aerobic exercise (20 min—3x/week—12 weeks) increases the levels of BDNF in the cargo of circulating extracellular vesicles Barcellos et al. (2020)	MDD patients have lower serum and exosomal levels of BDNF Gelle et al. (2021)
	Vascular endothelial growth factor (VEGF)	VEGF have been identified as an sEV cargo molecule in various peripheral tissues Zhang and Xu (2022). VEGF is increased by acute systemic exercise (1 h exercise bout at 50% of VO_2 max)_ in human skeletal muscle (Gavin et al., 2004)	Individuals who have attempted suicide show reduced levels of VEGF in their cerebrospinal fluid. Additionally, there is a negative correlation between the levels of VEGF and the severity of depression. Isung et al. (2012)
Cytokines	Interleukin (IL)-1β	Aerobic exercise (20 min—3x/wk—12 weeks) increases the levels of IL-1β in the cargo of circulating extracellular vesicles Barcellos et al. (2020)	Chronically elevated levels of IL-1β have been associated with the pathophysiology of MDD (Farooq et al., 2017)
	Interleukin (IL)-10	Exercise training (45 min/d x 7 days - aerobic 70% of VO_2 max_ + resistance) changes skeletal muscle small EV microRNAs that target inflammatory pathways such as IL-10, suggestive of reduced inflammation Su et al. (2022)	Some studies have shown that individuals with depression may have lower levels of IL-10, suggesting that reduced anti-inflammatory activity could be involved in the development of depressive symptoms (Roque et al., 2009). However, there is also evidence suggesting that higher levels of IL-10 could be linked to chronic or recurrent depression. (Roque et al., 2009)

## Conclusion and future directions

Aerobic exercise is a promising and evidence-based treatment for MDD, and the discovery that exercise-induced sEVs can promote intercellular communication by delivering cargo, acting on neighboring and distant cells, promoting healthier physiological status is an important new advance in the field of exercise. Knowing that both, the effects of exercise and the prevalence of MDD are influenced by sex, sex as a biological variable should be taken into consideration in future studies. The role of sEVs in the context of exercise and depression has yet to be investigated, however, based on the evidence summarized here, we identify common biological features between exercise and depression, exercise and sEVs, and sEVs and depression. We suggest that exercise’s effect on depression is mediated by altered peripheral sEV cargo signatures that influence neural pathways associated with the etiopathogenesis of major depression, such as neural plasticity and neuroinflammation. Future studies, including examining the effects of exercise-induced sEV cargo on depression and better understanding the mechanisms of bi-directional sEV transfer, as well as investigating the extent and type of sEVs released in different types, intensity, duration, and frequency of exercise, would provide important missing links across all three research domains.
